# The perception of odor objects in everyday life: a review on the processing of odor mixtures

**DOI:** 10.3389/fpsyg.2014.00504

**Published:** 2014-06-02

**Authors:** Thierry Thomas-Danguin, Charlotte Sinding, Sébastien Romagny, Fouzia El Mountassir, Boriana Atanasova, Elodie Le Berre, Anne-Marie Le Bon, Gérard Coureaud

**Affiliations:** ^1^Centre des Sciences du Goût et de l’Alimentation, CNRS UMR6265, INRA UMR1324, Université de BourgogneDijon, France; ^2^Smell and Taste Clinic, Department of OtorhinolaryngolyTU Dresden, Dresden, Germany; ^3^INSERM U930, Université François RabelaisTours, France; ^4^Unilever R&D, VlaardingenNetherlands

**Keywords:** odor mixture, perception, interactions, configural, elemental, animal behavior, human applications

## Abstract

Smelling monomolecular odors hardly ever occurs in everyday life, and the daily functioning of the sense of smell relies primarily on the processing of complex mixtures of volatiles that are present in the environment (e.g., emanating from food or conspecifics). Such processing allows for the instantaneous recognition and categorization of smells and also for the discrimination of odors among others to extract relevant information and to adapt efficiently in different contexts. The neurophysiological mechanisms underpinning this highly efficient analysis of complex mixtures of odorants is beginning to be unraveled and support the idea that olfaction, as vision and audition, relies on odor-objects encoding. This configural processing of odor mixtures, which is empirically subject to important applications in our societies (e.g., the art of perfumers, flavorists, and wine makers), has been scientifically studied only during the last decades. This processing depends on many individual factors, among which are the developmental stage, lifestyle, physiological and mood state, and cognitive skills; this processing also presents striking similarities between species. The present review gathers the recent findings, as observed in animals, healthy subjects, and/or individuals with affective disorders, supporting the perception of complex odor stimuli as odor objects. It also discusses peripheral to central processing, and cognitive and behavioral significance. Finally, this review highlights that the study of odor mixtures is an original window allowing for the investigation of daily olfaction and emphasizes the need for knowledge about the underlying biological processes, which appear to be crucial for our representation and adaptation to the chemical environment.

## INTRODUCTION

The way human beings map their environment as a brain representation is a cornerstone to the interactions they can develop with their surroundings and thus determines their fitness to the world they live in. This representation is built on the basis of sensory cues provided by sensory organs and gathered in the brain. The environment is particularly rich in volatile chemical compounds emitted from a large variety of natural and unnatural sources (e.g., plants, food, conspecifics, organisms, perfumes, human activities). The olfactory system must compute this mixture of volatiles, all day long at a certain distance from the sources and in a timescale reconcilable with fast but relevant behaviors. This is the challenge of the sense of smell, which has to extract relevant information from highly complex chemical mixtures. For humans and other organisms, the success of this computation is a prerequisite to a reliable mental representation of the olfactory environment, which is essential for maximizing adapted behaviors throughout life. Conversely, impaired olfactory processing may affect health and/or well-being and can even lead to death in certain species.

Efficient processing of odorants mixtures should allow for not only the instantaneous recognition and categorization of smells but also the discrimination of odors among others (e.g., background). The different ways in which the olfactory system processes an odor mixture relative to its components contributes to this discrimination. Nevertheless, though olfaction has been the subject of numerous studies, most of them used so-called “monomolecular odors” (i.e., they were based on single odorants as stimuli). As a consequence, the psychophysiological and neurobiological mechanisms that govern the perception of complex odor stimuli, namely the daily functioning of the sense of smell, remain poorly understood. In this context, the present review aims to depict the current knowledge on the perception of odor mixtures. The main guideline of this review is to gather and discuss the results of very recent as well as major studies on the processing of odor mixtures whatever they focused on cellular, neurobiological, behavioral or psychological aspects, and to take into consideration studies conducted both in humans and animals. Considering that olfactory neuroanatomy is remarkably conserved among animals (Ache and Young, 2005), we especially took advantage of studies in non-human species to highlight the ongoing research on the mechanisms of peripheral and central processing specific to complex odor stimuli. Then we discuss the implications of these mechanisms in relation to the perception of odor objects and the cognitive and behavioral significance of such a processing. Finally we consider the applied consequences and benefits that research on odor mixture perception may have for clinical approaches in individuals with mood disorder and for formulation approaches in the field of flavors and fragrances.

## THE SPECIFICITY OF ODOR MIXTURES PROCESSING: PERCEPTUAL INTERACTIONS

The main features of monomolecular odor processing are well characterized. Odor intensity is mainly driven by the odorant concentration (Stevens, 1960; Berglund et al., 1971; Chastrette et al., 1998; Devos et al., 2002). Odor quality is mainly related to the odorant chemical structure (Chastrette, 1997; Gaudin et al., 2007; Sanz et al., 2008; Kaeppler and Mueller, 2013; Snitz et al., 2013). Odor pleasantness is highly correlated to odor quality (Kermen et al., 2011) and largely depends on the molecular structure (Khan et al., 2007); odor intensity (Doty, 1975) and individual cognitive factors (e.g., Rouby et al., 2009) also impact pleasantness. However, in the case of odor mixtures, everything becomes more complicated due to the perceptual interactions that arise from the complex chemical signal encoding and processing within the olfactory system.

**FIGURE 1:**
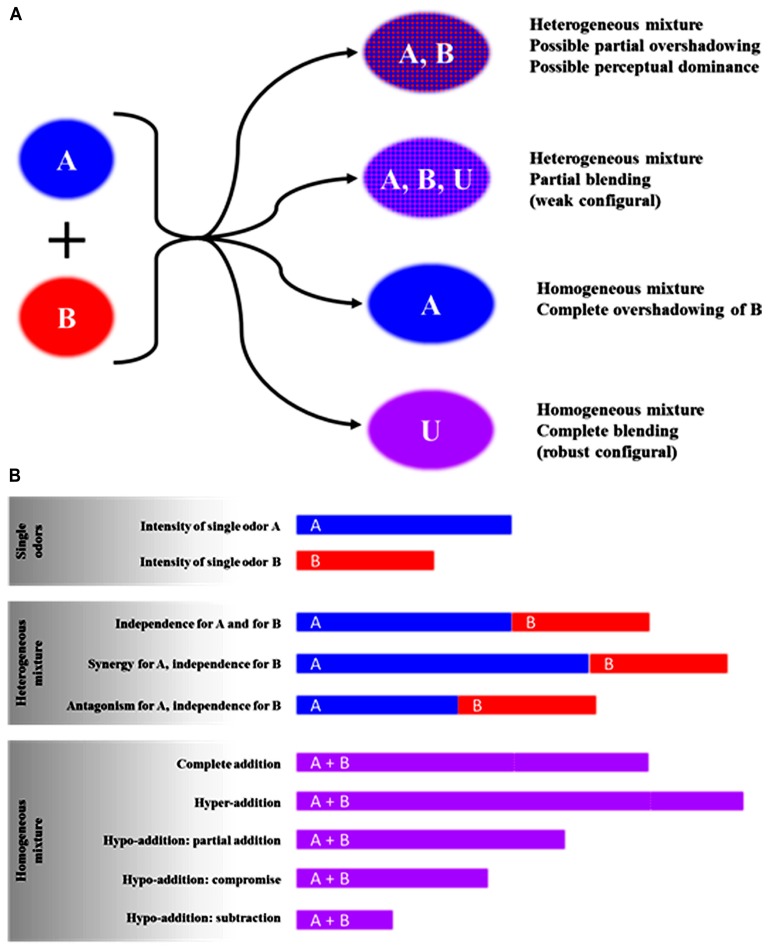
**Theoretical outcomes on odor quality (A) and odor intensity (B) when two odorants are perceived in the mixture.** One odorant has an odor noted A and the other B, while odor U is specific to the mixture (Unique-cue, see section on configural processing of odorants in mixtures) (this figure was partially adapted from Thomas-Danguin (1997).

As defined by Berglund et al. (1976), a mixture percept can be homogeneous when a single odor is perceived from the mixture or heterogeneous when several odors are perceived from the mixture. A homogeneous percept first arises when the odors of the mixed odorants blend into a new odor perceived as an entity. In that case, the mixture is called a blending mixture (Thomas-Danguin et al., 2007) and the perception may be considered configural (or robust configural; Kay et al., 2005) or synthetic (Berglund and Olsson, 1993; Laing, 1994). Second, the odor mixture could also be considered homogeneous when one mixture component has a strong intensity and thus completely covers the quality of the other components; in that case, one speaks about complete overshadowing (Kay et al., 2005) or masking (Cain and Drexler, 1974). When the percept induced by the mixture is heterogeneous, at least some of the component odors can be perceived within the mixture. This refers to the analytical processing of olfactory information (Berglund and Olsson, 1993) also qualified as elemental (Kay et al., 2005). In that case, the odor quality of the mixture can be predicted based on the odor intensity of the components (Laing and Willcox, 1983; Olsson, 1998; Wise and Cain, 2000), but some perceptual interactions may be observed, such as perceptual dominance or partial overshadowing (Atanasova et al., 2005a; Kay et al., 2005; Brodin et al., 2009; Kurtz et al., 2009; Ferreira, 2012b). In many cases, the mixture can have blending properties that lead to the perception of a specific odor for the mixture, on top of the odors of the odorants, which are still perceived (weak configural; Kay et al., 2005). **Figure 1A** illustrates all of the theoretical interactions for odor quality in binary mixtures. In the case of more complex mixtures, it has been suggested that the odor quality of the mixture is more frequently different from the quality of their constituting odorants. In other words, complex mixtures are more inclined to evoke the perception of a new odor (Livermore and Laing, 1998b; Ferreira, 2012b; Lindqvist et al., 2012).

Regarding odor intensity, perceptual interactions induced by the mixing of at least two odors can lead to several effects that can be categorized depending on whether the mixture quality is homogeneous or heterogeneous (Cain and Drexler, 1974; Berglund et al., 1976; Thomas-Danguin, 1997; Ferreira, 2012a; Thomas-Danguin and Dumont, 2012). To demonstrate the perceptual effect of mixing odors, the mixture intensity is compared to the intensities of the single components or their sum (Cain, 1975; Patte and Laffort, 1979; Berglund and Olsson, 1993; Thomas-Danguin and Chastrette, 2002); all of the theoretical possibilities are summarized in **Figure 1B**. For homogeneous percepts, hyper-addition, complete addition, or hypo-addition can arise. In the case of hypo-addition, depending on whether the mixture intensity is higher or lower than the single components’ odor intensities, one can observe partial addition, compromise, or subtraction (**Figure 1B**). In the case of heterogeneous percept, it is possible to differentiate among synergy, independence, or masking (partial overshadowing, **Figure 1B**). In the case of complex mixtures including more than two odorants, the odor intensity of the mixture usually does not increase when increasing the number of components (Berglund, 1974; Laffort and Dravnieks, 1982; Miyazawa et al., 2009; Ferreira, 2012a).

Pleasantness is another key feature of odors, but the perceived pleasantness of mixtures has been poorly studied. The available results on binary mixtures all suggest that the pleasantness of the mixture falls between the pleasantness of the components (Moskowitz and Barbe, 1977; Dravnieks and Jarke, 1980). More recently, it was reported that components’ odor intensity strongly contributed to the overall mixture pleasantness (Lapid et al., 2008). However, for greater than binary-order mixtures, pleasantness seems to be hardly predictable (Lindqvist et al., 2012).

Perceptual interactions induced by the perception of odorants’ mixtures could arise from several biochemical or neurobiological interactions during all stages of olfactory information processing within the olfactory system from the periphery to the brain, as reviewed hereafter.

## INTERACTIONS AT THE PERIPHERY: CODING COMPLEX CHEMICAL INFORMATION

Interactions occurring at the peripheral level of the olfactory system play a critical role in the processing of odorants’ mixture (Berglund et al., 1976; Bell et al., 1987; Derby, 2000; Kay et al., 2003; Goyert et al., 2007). In both vertebrates and invertebrates, the periphery of the olfactory system triggers the first step of olfactory information coding. At this stage, odorants are sampled by a large number of olfactory receptors (ORs) located in the cilia of olfactory sensory neurons/cells (OSNs). In mammals, each OSN expresses only one functional OR (Chess et al., 1994; Malnic et al., 1999; Serizawa et al., 2004), while insect OSNs express a conventional ligand-binding OR together with OR83b, a highly conserved member of the insect OR family (Larsson et al., 2004). Each OSN/OR typically responds to a variety of odorants so that the identity of a molecule is encoded by the combination of ORs/OSNs that recognize it (Malnic et al., 1999; Duchamp-Viret et al., 2000; Kajiya et al., 2001). The overlapping response profiles of OSNs introduce the possibility of interactions within the context of odorants’ mixtures.

Electrophysiological studies in different vertebrate and invertebrate species have compared the responses of OSNs to binary mixtures and their components (Ache et al., 1988; Caprio, 1989; Akers and Getz, 1993; Kang and Caprio, 1997; Steullet and Derby, 1997; Carlsson and Hansson, 2002; Ochieng et al., 2002; Duchamp-Viret et al., 2003). Three types of interactions were mainly observed; they depended on the odorants included in the mixtures and their concentration ratios. In many cases, the response intensity of OSNs to the mixture is lower than the response to the most efficacious component. This phenomenon is reconcilable with the compromise or the subtraction levels of hypo-addition (**Figure 1B**; Gleeson and Ache, 1985; Ache, 1989; Steullet and Derby, 1997; Duchamp-Viret et al., 2003; Rospars et al., 2008). Conversely, the response intensity of OSNs to a mixture can be higher than that induced by the most efficacious component; this phenomenon is classified as partial addition or hyper-addition when the response to the mixture exceeds the summed responses to the components (**Figure 1B**; Akers and Getz, 1993; Kang and Caprio, 1997; Ochieng et al., 2002; Duchamp-Viret et al., 2003; Chaput et al., 2012). In most cases, a given type of interaction was observed over the whole concentration range, but in some cases, a shift to another interaction type as a function of odorant concentration was reported (Duchamp-Viret et al., 2003; Rospars et al., 2008). Data modeling suggests that both competitive and non-competitive interactions occur at the OR level and may account for the effects reported in these studies (Rospars et al., 2008; Cruz and Lowe, 2013; Münch et al., 2013). There is competitive interaction when two molecules bind to the same receptor binding site. This mechanism could involve either two agonist odorants, i.e., molecules that are able to activate the receptor, or one agonist and one antagonist (the latter being a molecule that binds to the receptor but is unable to activate it; Spehr et al., 2003; Oka et al., 2004; Sanz et al., 2005, 2008; Jacquier et al., 2006). For example, it has been shown that the odorant bourgeonal is a powerful agonist for the human receptor hOR17-4 recombinantly expressed in human embryonic kidney (HEK) 293 cells, while another odorant undecanal fails to activate this receptor (Spehr et al., 2003). However, the co-incubation of bourgeonal with undecanal strongly suppressed the hOR17-4 response, which indicates that undecanal inhibited the receptor activation by bourgeonal. The electrical activity in the human olfactory epithelium in response to bourgeonal was dramatically decreased after undecanal exposure (Spehr et al., 2004). Moreover undecanal odor exhibits a strong inhibitory effect on bourgeonal odor at the perceptual level in humans (Spehr et al., 2004; Brodin et al., 2009). A recent study (Chaput et al., 2012) gave additional evidence for a direct link between peripheral and perceptual responses to a mixture containing two odorants naturally occurring in wine, i.e., whiskey lactone and isoamyl acetate. Rat OSN responses to this mixture were enhanced or reduced depending on the OR type and/or the concentration of whiskey lactone in the mixture. Similarly, in humans, the fruity note intensity within the same mixture was increased by low concentrations of whiskey lactone and decreased by high concentrations. Thus, for a given mixture, different types of interactions can occur at the peripheral level, depending on the odorant concentration ratios, which likely govern the mixture’s perceptual properties. In insects too, various types of interactions occur at the periphery after stimulation with mixtures of plant odorants and pheromones (Ochieng et al., 2002; Deisig et al., 2012). Hypo-addition-like effects have been observed in a number of cases, and inhibition caused by one molecule at the level of OSNs can modify the response to a pheromone either by reducing its magnitude or by modifying its temporal dynamics (Su et al., 2011; Deisig et al., 2012).

Overall, studies in vertebrates and invertebrates highlight the importance of peripheral interactions in the coding of odorants’ mixtures. These events likely shape the odor signal, which might determine the perceptual features of complex mixtures. Nevertheless, the peripheral coding of odorants’ mixtures remains poorly understood, and it is still difficult to predict the outcomes of this process though the properties of the single compounds are known.

## INTERACTIONS AT HIGHER LEVELS: PROCESSING ODOR INFORMATION

The emergence of new methods of brain imaging in both humans and animals has shed new light on how odors, especially those elicited by mixtures, are encoded in the brain olfactory regions where activation or inhibition between neurons or clusters of neurons can occur. From an anatomical point of view, the OSN enters the olfactory bulb (OB, mammals) or antennal lobe (AL, insects) and connects the mitral cells (mammals) or projection neurons (insects). In mammals, OSNs expressing the same OR converge onto one glomerulus and connect one mitral cell, which is accompanied by tufted cells (Buonviso and Chaput, 1990; Mombaerts et al., 1996; Chen et al., 2009). In insects, similar OSNs also converge onto one glomerulus (Galizia and Menzel, 2000; Wang et al., 2003), but one glomerulus can connect several projection neurons (Kirschner et al., 2006). This neuronal architecture helps gather information from several similar OSNs while staying close to the combinatorial code provided by the binding odorant/OR. Nevertheless, inhibitory systems at this brain processing level can modify the output information that is projected to higher areas. A significant modification of the odor output code occurs post-synaptically and is triggered by granular cells in mammals (Wright and Smith, 2004; McGann et al., 2005; Kay and Stopfer, 2006; Abraham et al., 2010). In insects, inhibition arises from local neurons that connect glomeruli pre- and/or post-synaptically (Silbering and Galizia, 2007).

In odorants’ mixture processing, perceptual interactions occurring at the OB/AL level are thought to mostly result from these inhibitory processes, which may contribute to the sparse representation of complex odor mixtures in these brain structures (Dulac, 2006). This may also lead to the apparent perceptual contribution of only a few dominant chemical cues within a complex mixture (e.g., natural scents; Dulac, 2006; Clifford and Riffell, 2013). In line with the involvement of inhibitory processes in the OB, it has also been reported that mitral/tufted cells respond to odorants presented both individually and in mixtures, although the firing rates evoked by mixtures typically showed partial suppression (i.e., hypo-addition; **Figure 1B**; Davison and Katz, 2007). However, an unanswered question is what triggers the inhibition. One hypothesis is that chemical (structural) similarity between odorants could activate overlapping patterns, which may induce perceptual similarity but may also increase the interaction potential (Linster et al., 2001; Grossman et al., 2008). Indeed, at a behavioral level, rats discriminate a binary mixture from its components better when the components are perceived as very similar (Wiltrout et al., 2003). Using a computational model Linster and Cleland (2004) went further and showed that mixing odorants with similar glomerular patterns resulted in lateral inhibition in the OB that lead to a loss of information about each single odorant. This loss of information would favor a bulbar pattern of activation specific to the mixture and contribute to a distinct code for the mixture compared to the code of each component, in line with configural processing of the mixture (but see Fletcher, 2011). However, an alternative theory was proposed to account for these results and suggests that very overlapping odorants, in terms of glomerular activation pattern, would not induce a configural perception because of their almost perfect perceptual similarity (Frederick et al., 2009). Thus, a concentration effect may be considered: mixing two odorants that are perceptually similar would be like doubling the concentration of one odorant. The change in concentration can actually modify the quality of the odor (Laing et al., 2003).

Interactions also occur at the AL level in insects. In the honeybee, the glomerular pattern activated by hexanol and citral in a mixture is different from the sum of patterns activated by each odorant (Joerges et al., 1997). This difference supposedly results from the activation/inhibition of close glomeruli via local neurons, not from the odorants’ similarity (hexanol and citral are not structurally or perceptually similar), even if, as proposed in mammals, configural processing is more likely to occur in mixtures of similar odors (Deisig et al., 2002). In this species, the pre-synaptic transduction of information appears to be mainly ruled by elemental laws (Deisig et al., 2006). In contrast, because of lateral inhibition, the output from the AL to higher-order brain regions by projection neurons supports a more configural and less elemental type of processing (Deisig et al., 2010); patterns sent to superior areas would directly encode configurations. In sum, at the OB/AL processing level, lateral inhibition and mixture-specific cell activation were observed and could account for the perceptual interactions induced by the processing of odor mixture.

Beyond these primary brain structures, the olfactory information is processed in superior areas of the brain. In mammals, mitral cells project to the anterior olfactory nucleus, anterior and posterior piriform cortex (aPC and pPC), olfactory tract, lateral entorhinal cortex, and part of the amygdala, among other regions (Mori and Sakano, 2011). The piriform cortex (PC) has been the center of several investigations related to odor discrimination and representation, some of which have used mixtures of odorants (Haberly and Bower, 1984; Granger and Lynch, 1991; Litaudon et al., 1997; Haberly, 2001; Wilson and Stevenson, 2003a; Kadohisa and Wilson, 2006; Barnes et al., 2008; Howard et al., 2009; Stettler and Axel, 2009; Bekkers and Suzuki, 2013). The processing of olfactory information in the OB and the PC is highly contrasted. A study of odorants’ mixture processing in mice revealed nonlinear combinatorial interactions at the PC level, as shown by a broader responsiveness of the anterior PC neurons relative to the OB mitral cells (Lei et al., 2006). From a functional point of view, it has been shown in rats that the PC can rapidly discriminate a mixture from its components, thereby producing a minimal cross-habituation to components after habituation to the mixture, while the OB still computes the mixture like the sum of odorants (Wilson, 2000, 2003). Because, the aPC and pPC are quite different in their anatomical organization, they likely have distinct roles in odor encoding: encoding of odorant identity may occur in the aPC while encoding of odor similarity or odor quality occurs in the pPC (Kadohisa and Wilson, 2006; Yoshida and Mori, 2007). These dissociated roles of the aPC and pPC were confirmed by a functional magnetic resonance imaging (fMRI) study performed in humans with single odorants (Gottfried et al., 2006; Gottfried, 2009; Howard et al., 2009). When taken together, these results suggest that the pPC is a key structure for the perception of odor mixtures since it may contribute to their configural processing, namely their putative coding as odor objects, each carrying a specific odor quality.

Higher-order cortices are also involved in olfactory information integration. In a positron emission tomography (PET) study comparing the brain processing of citral + pyridine mixtures, the odors of the single odorants and mixtures both activated the primary and secondary olfactory regions. However, the contrast between the two types of stimuli revealed activation in the middle cingulate cortex, superior frontal gyrus, and angular gyrus (Boyle et al., 2009). In this study, the lateral and anterior regions of the OFC played a distinct role in mixtures’ processing and responded in a preferential manner to the binary mixtures. The anterior portion of the OFC acted such as an on-off detector for odor mixtures because it was activated in response to odor mixtures and deactivated in response to single odorants; the lateral portion of the OFC responded in a graded fashion to relatively small differences in intensity ratios of the two mixed odors (Boyle et al., 2009). Anatomically, the OFC is located at a three-synapse step from the olfactory epithelium and receives information already computed by the OB and PC/amygdala (Gottfried and Zelano, 2011). This cortex is known to encode odor identity (quality) but also odor valence (Anderson et al., 2003; Royet et al., 2003) and odor significance (acquired value; Critchley and Rolls, 1996). Therefore, this structure probably plays a major, but still unknown, role in the configural processing of complex odor stimuli. A contrasted processing of binary odor mixtures and their single odorants was also observed by fMRI in higher-order brain areas but not primary olfactory cortices (Grabenhorst et al., 2007). In this study, different parts of the OFC simultaneously and independently represented the positive and negative hedonic value of an odor mixture that contains pleasant and unpleasant components. Interestingly, the medial OFC responded more to the jasmine’s pleasant odor when it is mixed with a small amount of the unpleasant odor of indole (Grabenhorst et al., 2007). This response may reflect the perceptual synergy or pleasantness enhancement of the pleasant odor sometimes observed when mixed with an unpleasant one. Such perceptual outcome could be due to an attention-capturing effect of hedonically complex mixtures that operate unconsciously and involve the superior frontal gyrus (Grabenhorst et al., 2011).

## ODOR OBJECTS: CONFIGURAL PROCESSING OF ODORANTS IN MIXTURES

Perceptual interactions induced by the previously reviewed neurobiological mechanisms can be considered as an effectiveness of the olfactory system to capture the complex chemical information as a whole or as elements pertaining to the whole. Indeed, in both mammals and insects, these perceptual interactions are the basis of configural and elemental processing of mixtures of odorants, which may lead to the perception of mixtures as odor objects (configurations) or not. This section of our review focuses on the results that support the notion of odor objects perception.

### THE LIMIT IN ODOR MIXTURES ANALYSIS

A key finding supporting the odor object theory is the number of odorants that can be discriminated and identified within an odorants’ mixture. This is most likely one of the most investigated question in the human perceptual analysis of odor mixtures (Laing and Francis, 1989; Laska and Hudson, 1992; Jinks and Laing, 1999a,b; Laing and Jinks, 2001). The resolution of this central question should give cues about odorants (or odors) that primarily contribute to the global mixture’s percept. A series of studies have shown that humans are hardly able to identify more than three odorants in a mixture that contains up to eight odorants (Laing and Francis, 1989; Laing and Livermore, 1992). This limitation is not a function of the stimulus features. Indeed, untrained subjects cannot correctly identify more than four familiar odors in a mixture containing up to eight odorants (Livermore and Laing, 1998b). Trained subjects reach the same odor identification limit when submitted to mixtures of familiar odors issued from a complex composition designed to evoke real odor sources (e.g., lavender, cheese; Livermore and Laing, 1998a). Cognitive factors play a minor role in the human in-mixture odor identification limit. Focusing subjects’ attention on a specific quality to be identified in a mixture containing up to six odorants does not increase the identification rate compared to the standard identification task, in which all odors have to be identified (Laing and Glemarec, 1992). Moreover, training or expertise does not enhance the identification performance since only three or four components of a mixture containing up to five odorants can be correctly identified by either a trained panel or an expert panel (Livermore and Laing, 1996).

Considering these results, the group of D. G. Laing concluded that the human limit of identification of in-mixture odors may be imposed physiologically or by processing constraints. Even in binary mixtures, there might be a loss of the odorant’s major characteristic because of inhibitory interactions within the olfactory processing pathway, especially in the OB as reviewed above, or by a limit in working memory, which likely impairs identification. Similar findings were reported in animal studies. Adult rats have difficulty identifying components within mixtures with more than three or four components (Staubli et al., 1987), but many odorants in a mixture can be more readily identified by honeybees (e.g., Reinhard et al., 2010). The interpretation of this compilation of more than 10 years of research appears to be in line with the hypothesis of configural functioning of olfaction, which is analogous to that for facial and object recognition (Jinks and Laing, 2001).

### THE CONCEPT OF ODOR OBJECTS

Odor object recognition would allow for the sense of smell to perform feature extraction and object synthesis that lead to the elaboration of a stable, background-detached representation of complex signals. Due to interactions within the olfactory processing pathway, a stereotyped map could be elaborated; this map, where odor identity can be represented in spatiotemporal patterns, may be specific to a given complex stimulus and contain information about the elements of the mixture and likely about their association. The unique spatial and temporal signature could be recognized in the brain as an entity against a background of other odors and identified as an odor object (Margot, 2009). To perform this complex task, the brain could rely on rapid and specific cortical adaptation to background odors and recognition of bulbar activation patterns (Stevenson and Wilson, 2007; Frank et al., 2010). When a stimulus activates the olfactory system, the activation pattern produced at the OB level, and further processed in cortical areas, would be compared to stored ones (for details about the processing mechanisms see the previous sections on interactions at the periphery and interactions at higher levels). If there is a good match, we consciously experience a discrete odor that is distinct from the background and discriminable from other odors (Stevenson and Wilson, 2007). If there is no match between the bulbar incoming pattern and a stored one, the novel pattern may be rapidly acquired (Stevenson and Wilson, 2007). Even if alternative definitions of odor objects have been proposed (Yeshurun and Sobel, 2010), suggesting a critical role of hedonic features, the most commonly accepted definition relies on the integration of a specific blend of volatile molecules that can be separated from the surrounding clutter of volatiles to stand out as an entity reflecting a putatively unidentified specific source (e.g., a melon’s odor in the market).

The principle of a unique spatial and temporal signature for complex odor stimuli, which accounts for odor object perception, is in line with configural processing of odorants’ mixtures. Following Rescorla’s unique-cue theory (Rescorla, 1972, 1973; Rescorla et al., 1985), an odor mixture can carry, beside the elements, another stimulus that is unique to the combination of those elements. In other words an AB binary mixture may be conceptualized as being composed of the individual A and B elements as well as a separate stimulus unique to the AB combination, usually noted U (unique-cue; **Figure 1A**). However there is an unresolved debate in the literature regarding the unique-cue theory and its consequences in complex stimuli configural learning experiments (Brandon et al., 1998; Harris, 2006). Indeed, from Rescorla’s point of view, in a conditioning paradigm one can learn about the separate elements A and B but also U, and the associative strength of U is then equal to the sum of the strengths of the elements. The unique-cue stimulus is thought to occur at the level of memory representation rather than that of perceptual representation or spontaneous processing (Rescorla et al., 1985). Adopting a different point of view, Pearce’s configural approach (Pearce, 1987, 1994) proposes that the unique stimulus, U, which is specific to the mixture, is represented as a configural pattern whose elements are integrated prior to any learning. Whether Rescorla’s or Pearce’s view of configural learning better accounts for experimental results is not resolved yet (Dreumont-Boudreau et al., 2006).

**FIGURE 2:**
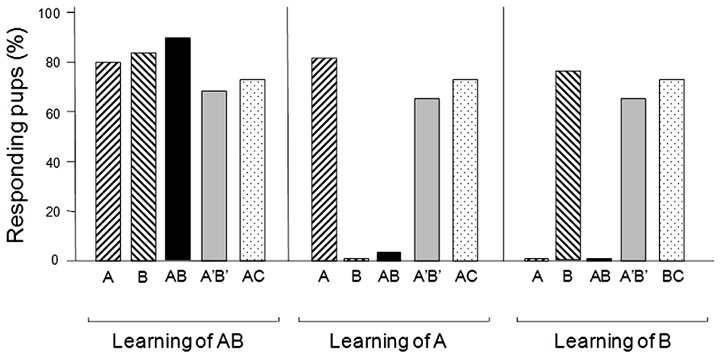
**Proportions (%) of 2- or 3-day-old newborn rabbits responding behaviorally (sucking response) to the odorant A (ethyl isobutyrate), the odorant B (ethyl maltol), their AB or A’B’ mixtures (respectively, at a ratio of 30/70 and 68/32 of the two components), and the AC mixture (C: guaïacol; ratio 50/50) after a single conditioning to the AB mixture or to one of its components.** The results show that after conditioning to AB, the pups respond both to the odorants and the different mixtures. Therefore they perceive the elements A and B during the learning episode. However, after conditioning to odorant A or B, they respond to the conditioned odorant but not to the AB mixture; nevertheless, they respond to the A’B’ and AC mixtures. Thus, newborn rabbits perceive the odor of a configuration in the AB mixture in addition to the odors of each odorant, while they perceive only (or mainly) the elements in the A’B’ and AC mixtures (adapted from Coureaud et al., 2008, 2009, 2011).

There are several lines of evidence showing that animals are able to perform configural processing of odor mixtures and thus differentiate between mixtures and their constituting monomolecular odors (insects: Chandra and Smith, 1998; Lei and Vickers, 2008; Riffell et al., 2009; Deisig et al., 2010; van Wijk et al., 2010; Riffell, 2012; Szyszka et al., 2012; aquatic animals: Derby et al., 1996; Valentincic et al., 2000; Tabor et al., 2004; mammals: Staubli et al., 1987; Kay et al., 2003; Wiltrout et al., 2003; Dreumont-Boudreau et al., 2006). This seems to be true even early in life. For instance, a binary mixture of ethyl isobutyrate and ethyl maltol is configurally processed, at least in part, by newborn rabbits. For the pups, this mixture spontaneously evokes an odor that is different from the one of its constituting odorants and provokes very contrasted behavior in a conditioning paradigm using the mammary pheromone (Coureaud et al., 2008, 2009, 2010, 2011; **Figure 2**). Similar results were obtained with a more complex mixture of six odorants (RC mixture; Sinding et al., 2013).

**FIGURE 3:**
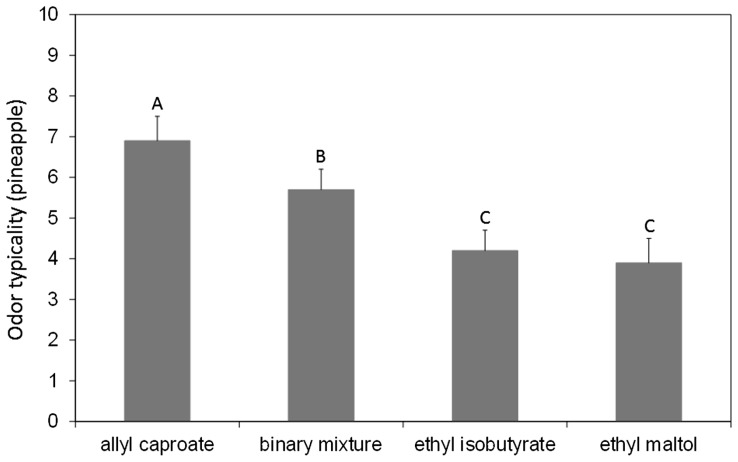
**Mean typicality ratings (gray bars) of the term pineapple obtained with a group of 20 untrained subjects for a binary mixture of ethyl isobutyrate and ethyl maltol, each single odorant and a control odorant (allyl caproate carrying a typical pineapple odor).** The error bars represent the standard error of the mean. The same letters indicate that the means were not different at a significance level of 5%. The results indicated that the binary mixture carried a pineapple odor that was significantly less present in the single odorants. This finding supports the idea that the odor quality of the mixture is different from those of its components (adapted from Le Berre et al., 2008b).

These results from animal studies demonstrate the possibility of specific encoding for odor mixtures compared to their constituting elements. However, it is worth noting that the nature of stimulus representation is inferred from experiments examining how the conditioned response to one odorant or a mixture of two or more odorants generalizes to another single odorant or mixture (Harris, 2006). As a consequence, whether the mixture configuration is reconcilable with odor object encoding is not straightforward in animal studies. One way to circumvent this issue is to address the question in parallel in animals and humans. In humans, even if configural processing is difficult to demonstrate, it is advantageously possible to assess whether an odor mixture has a different quality from its single odorants (Livermore and Laing, 1998a; Jinks and Laing, 2001; Bott and Chambers, 2006; Weiss et al., 2012; Chambers and Koppel, 2013). Following an animal/human parallel approach, we have shown that the binary mixture of ethyl isobutyrate and ethyl maltol used in rabbit pups (Coureaud et al., 2008, 2009, 2011) evokes, in human subjects, a more typical odor of pineapple (Le Berre et al., 2008b; Barkat et al., 2012) and is more frequently identified as a pineapple odor (Le Berre et al., 2010) compared to the single odorants (**Figure 3**). Similar results were obtained with the RC mixture of six components, which is configurally perceived by newborn rabbits and specifically evokes a red cordial odor in human adults (Le Berre et al., 2008b; Sinding et al., 2013). These findings, which resulted from the combined data obtained in rabbit pups and human adults, support the idea that mixtures of odorants can be perceived as odor objects in the sense that they can be configurally processed and can evoke new percepts, different from those of the elements, and which could be attributed to unique sources (e.g., pineapple or red cordial).

### THE CRITICAL IMPACT OF STIMULUS COMPOSITION

Natural chemical signals frequently undergo concentration changes that produce differences in both the level and pattern of activation of ORs. This variability makes the processing of complex stimuli even more difficult, since the olfactory system must extract perceptual constancy from inconstant input (Gottfried, 2010). It has been argued that complex stimuli recognition might be concentration-invariant and mostly results from ratio-information extraction (Cleland et al., 2007). For instance, rats can discriminate binary odor mixtures according to the molar ratios of their components, which further ensures mixture odor recognition at higher or lower concentrations (Uchida and Mainen, 2008). The ratio of odorants in binary odor mixtures was also found to be the driving factor for odor processing and perception in insects (e.g., Clifford and Riffell, 2013) and in catfish (Valentincic et al., 2000). In rats, a binary mixture of the same two odorants can be processed elementally, configurally, or induce overshadowing (Kay et al., 2003; McNamara et al., 2007). The impact of mixed odorants ratios was clearly observed at the OSN level in rats (Chaput et al., 2012). In humans, psychophysical studies have clearly shown that odorants’ ratio and, more precisely, odorants’ intensity proportions in a heterogeneous binary mixture, largely determine the odor quality perception (Olsson, 1994, 1998). Supporting these findings, data obtained in both rabbit pups and human adults demonstrate the influence of in-mixture odorant ratios on processing and perception. In rabbit pups, while a 30/70 ratio of ethyl isobutyrate and ethyl maltol induced the configural processing of the mixture, a reversed ratio (68/32) induced the elemental processing of this mixture (Coureaud et al., 2011; **Figure 2**). In human adults, a barely detectable variation of one odorant concentration in the same mixture (slight variation the ratio of the odorants), influenced its perception and particularly decreased its typicality toward pineapple (Le Berre et al., 2008a). A similar influence of the odorants’ proportions was observed with the more complex six-odorant RC mixture since a modification of the concentration ratio resulted in a significant shift in odor quality, which depended on the extent of the proportion modification (Sinding et al., 2013, 2014). Therefore, the odorant concentration ratio in a mixture is clearly a key factor that can drive the configural versus elemental perception of the mixture.

The chemical nature, or the odor quality, of the mixed odorants is another key factor of mixture processing (Kay et al., 2003, 2005). Indeed, it is well-established from human studies dealing with food aroma analyses that there are key compounds in the complex chemical mixture of volatiles responsible for a given food aroma (e.g., Escudero et al., 2004; Falcao et al., 2012). Studies in animals have also demonstrated that certain odorants in mixtures can be more readily identifiable than others (Staubli et al., 1987; Laska and Hudson, 1993; Kay et al., 2005; Reinhard et al., 2010). Therefore these odorants can contribute more strongly to the overall perceptual quality of the whole odor mixture. For instance, in rats, the identity of the odorant removed from a complex 10-component mixture affected the discrimination between the 10-odorant mixture and the nine-odorant sub-mixtures. Nevertheless, rats had difficulty discriminating the whole mixture from the same mixture with one component missing. These results suggest that the missing component was most often “filled-in” by the olfactory system to promote perceptual stability (Barnes et al., 2008; Chen et al., 2011; Chapuis and Wilson, 2012; Lovitz et al., 2012). In contrast, rats could reliably discriminate mixtures containing even small traces of contaminants from unadulterated complex mixtures; indeed, the replacement of an odorant by another was easily detected, and in a concentration-dependent manner (Wilson and Sullivan, 2011; Lovitz et al., 2012). Data obtained in newborn rabbits have shown that once conditioned to one of the odorants, whatever the odorant, animals cannot generalize their behavioral response to a six-odorant RC mixture configurally processed. This result supports the idea that the two stimuli are discriminated. Nevertheless, animals can generalize their response to the same mixture in which one odorant is missing (five-component mixture), regardless of the odorant (Sinding et al., 2013). These last results suggest that each odorant is a key odorant for rabbit pups. In contrast, data obtained using the same mixture in human subjects have shown that the red cordial odor quality carried by this six-odorant RC mixture is significantly different from the odor quality of some, but not all, sub-mixtures in which one odorant was missing (Sinding et al., 2013). Therefore, in human adults, many components would contribute more strongly to the overall perceptual quality of the odor mixture than do others. Even at subthreshold level, many odorants can modify the perception and/or the processing of odor mixtures (Atanasova et al., 2005b; Pineau et al., 2009; Lytra et al., 2012; Hummel et al., 2013).

Interestingly, it has been recently reported that different mixtures made of 30 equally intense, non-overlapping components that span the physicochemical space of odorants, give rise to a similar odor quality for humans. This finding lead the author to term such percept as an “olfactory white” (similar to a white color or “white noise”; Weiss et al., 2012). The need to equilibrate each component intensity in this study is reconcilable with the key role of the mixture ratio; however, the absence of a link between a single odorant’s odor quality and the mixture’s odor is at odd with the concept of key odorants in the perception of these specific mixtures. Even if such specific mixtures would be unlikely in ecological conditions, their processing is consistent with the concept of odor objects and might be of significant value as a model to decipher the mechanisms of odor mixture perception.

### THE IMPORTANCE OF INDIVIDUAL FACTORS

Individuals from the same species do not necessarily perceive the same odor in a particular odorant, and more generally, they do not present the same sensibility to odor cues (Amoore, 1967; Frumin et al., 2013). This inter-individual variability may result from many factors, e.g., genetic and/or anatomical differences; health status; ecological constraints; effects of experience; age and the abilities associated with the specific needs that characterize the successive stages of development; and semantic knowledge (in humans). For example, anosmia to certain odorants is shared between identical twins and transmitted to offspring (Wysocki et al., 1977; Wysocki and Beauchamp, 1984). Conversely, some individuals have a better sensitivity for certain odorants compared to other individuals (Keller et al., 2007; Menashe et al., 2007; Mainland et al., 2014). In this context, one may hypothesize that a contrasted sensitivity toward the components of a mixture can affect the ability to perceive odorants in mixtures and therefore directly influence the elemental vs. configural perception of the mixture. One may suggest that the ratio of the component thresholds drives the perception of the mixture by the subjects, as occurs with the ratio of concentrations. Such questions remain to be explored in detail, but preliminary results in human adults indicate that some subjects perceive the pineapple AB mixture in a more robust configural way than do others; curiously, the more the subjects have a configural perception of AB, the lower their detection thresholds of the components (Sinding, 2012; Sinding et al., in preparation).

Regarding developmental aspects, one may consider that due to the maturation of the sensory systems and brain and the change in ecological niches encountered by the organism over the development, the processing of odor mixtures may also be modified over time. In particular, around birth, the urgent need for neonates to acquire knowledge about the novel, aerial environment, could result in higher elemental abilities than in adults. Later in life, increased experience with a large variety of more or less complex odors and repeated exposure to some of the complex odors could promote their encoding as odor objects. While some results are in line with this developmental hypothesis (Sinding et al., 2013), others show that the perception of olfactory configuration is already present in young animals, and that neonate and adult mammals perceive certain mixtures of various chemical complexity in a comparable way (Coureaud et al., 2008, 2009, 2011; Sinding et al., 2013). This is consistent with the chemical complexity of early life environments (perinatal niches) from which organisms must rapidly extract salient information despite their immaturity, only relative (see the section dedicated to behavioral aspects below).

### THE IMPACT OF LEARNING

In addition to the previously discussed factors that clearly influence odor mixture processing, it is crucial to emphasize that the perception of odor mixtures is under cognitive control and that learning could shape this perception, but depending on the mixture. Perceptual learning, which contributes to the improvement of an organism’s ability to extract information from the environment (Gibson, 1969; Rabin et al., 1989), can affect the way in which a mixture of odorants is processed. In humans, odor-odor perceptual learning has been described and is likely comparable to odor-taste learning (Wilson and Stevenson, 2003a; Case et al., 2004; Stevenson et al., 2007). For instance, when two odorants were repeatedly experienced in a binary mixture, each odorant’s odor could acquire the perceptual quality of the other. This was demonstrated in a study in which an odorant, initially perceived with a cherry odor, smelled smokier after having been repeatedly experienced in mixture with guaiacol, another odorant perceived with a smoky odor. Furthermore, guaiacol smelled more like cherry after the co-exposure (Stevenson, 2001a). Odor-odor learning is not just stimulus -or quality- specific but is also a direct consequence of the learning procedure (Stevenson, 2001a). Odors experienced in a mixture were judged to be more alike than were odors smelled an equal number of times but out of mixture. This exchange of perceptual qualities between mixed odorants is related to how similar the elements were judged (Stevenson, 2001a). These results support the idea that the representation of odor qualities can combine to form new configurations that carry their own odors. These results also indicate that cognitive processes are engaged to decrease the chemical complexity of the environment by building experience-dependent perceptual associations (Wilson and Stevenson, 2003a).

Results obtained in animal studies also demonstrate the impact of conditioning on odor mixture processing (Livermore et al., 1997; Valentincic et al., 2000; Gerber et al., 2011). For instance, one conditioning experience to the previously mentioned mixture of ethyl isobutyrate and ethyl maltol (which smells like pineapple to human adults) allowed rabbit pups to generalize their response to both odorants, something they cannot do when tested with the mixture after single conditioning to one odorant only (Coureaud et al., 2008, 2009; **Figure 2**). However, repeated conditioning to this binary mixture led to a drastic decrease of generalization and the pups became more responsive to the mixture than to the elements. This result suggests an improved configural perception of the mixture. Conversely, after repeated conditioning to a single component, the pups responded to the mixture, which suggests improved elemental perception. Interestingly, these perceptual changes greatly depend on the mixture and its components. Indeed, with a mixture of ethyl isobutyrate and guaïacol, the same paradigm of repeated conditioning had no consequence on the perception, and the mixture remained always elementally perceived (Sinding et al., 2011). These results suggest that the initial status of the mixture, either purely elementally processed or akin to configural perception (i.e., weak configural; **Figure 1A**), likely plays a critical role in further cognitive processing.

Perceptual experience can also be acquired by passive exposure to odors (Rabin, 1988). When the olfactory environment of rats was enriched, their ability to discriminate odorants in binary mixtures increased (regardless of the odorant to which the rat was exposed during the enrichment period; Mandairon et al., 2006b,c). This effect was linked to neurogenesis in the rat OB (Mandairon et al., 2006a). In human adults, the mixture of ethyl isobutyrate and ethyl maltol was less configurally processed by a group of subjects after passive exposure to the single elements compared to non-exposed subjects. Perceptual learning would then favor the elemental perception of the mixture (Le Berre et al., 2008b).

Expertise is also a cognitive factor that can influence odor mixture perception. In a typicality rating task, experts in oenology rated the pineapple typicality of the ethyl isobutyrate and ethyl maltol mixture as equivalent to that of ethyl isobutyrate, while naïve participants rated this typicality as significantly higher compared to both elements perceived out of the mixture (Barkat et al., 2012). Thus, experts would be less sensitive to the configuration induced by the mixture. One could hypothesize that due to their perceptual expertise acquired through training to single odors, experts may be more inclined to focus on the elements’ odor in the mixture, which may make them more efficient in elemental processing. The ability to focus on the elements may be linked to their familiarity with the odorants, insomuch that the identification ability increases when the target is familiar (Rabin, 1988; Rabin et al., 1989). In this regard, identifying a familiar target mixed with a familiar contaminant was found to be easy (87% correct identification), while finding an unfamiliar target mixed with an unfamiliar contaminant was much more difficult (58% correct identification; Rabin et al., 1989). Nevertheless, learning, considered as perceptual training in experts, increases the absolute ability to identify odors in low but not highly complex mixtures. Indeed experts were more proficient than non-experts at discriminating and identifying odors in binary and ternary mixtures; for quaternary mixtures the correct identification rate fell below 20%, regardless of the expertise level (Livermore and Laing, 1996).

Expertise can also rely on semantic knowledge (Rabin, 1988; de Wijk and Cain, 1994; Stevenson, 2001b), which is another cognitive factor that influences odor mixture processing in humans. In a dedicated experiment assessing the impact of semantic learning on the perception of odor mixtures, it was found that exposure to the mixture target odor label (semantic learning) facilitated the perception of the configural odor of blending mixtures (Le Berre et al., 2010). Thus, verbal labels could have provided perceptually expected and reliable information regarding the frame of reference for odors (Herz and von Clef, 2001; Rouby et al., 2005), which may result in the top-down facilitation of odor recognition. A similar cognitive top-down effect, even if not directly related to semantic knowledge, could explain the results obtained in a study exploring the influence of odor context on odor mixture perception (Arao et al., 2012). Using colors that are congruent with the odor of each element of a binary mixture, it has been shown that participants judged the odor of the element congruent with the color to be more dominant in the mixture. The visual cue could have directed the participants’ attention toward the color-congruent odor, which then led to an enhancement of its perceptual representation within the mixture. In line with attentional processes, perceptual processing strategies may also modify odor mixture perception. The same blending mixture was less configurally processed by a group of naïve subjects engaged in an analytical task compared to a group of subjects engaged in a configural task (Le Berre et al., 2008b).

Taken together, these results demonstrate that odor mixture perception can be modulated by cognitive and/or attentional factors. According to the high complexity of the environment, it is likely that learning and attention can fine-tune the perception by highlighting the meaningful elemental features or configural shapes from the background (Wilson and Stevenson, 2003b).

## IMPLICATIONS OF ODOR MIXTURE PROCESSING ON BEHAVIOR

In the real life situation, odors are important vectors of information that elicit behavioral decisions from animals in their natural environment. For instance, odors are involved in the interaction between conspecifics, with competitors and predators, and in the selection of habitats, preys and food. Odors are never perceived alone, but among other odors, and chemical mixtures are usually the global stimuli that drive chemically mediated patterns of animal behaviors. Therefore, animals have no choice but to simplify the surrounding amount of information, which constantly varies over time. They must adapt to the chemical complexity of the environment by extracting information from this mass of molecules, especially in mixtures, by discriminating and assigning meaning to some of them and responding in a way adapted to their needs.

One strategy to reduce this complexity is to respond to certain odorants among others present in the same mixture, i.e., to focus on elements triggering behavioral responsiveness by themselves. This occurs when organisms respond to key odorants in complex odorous substrates, e.g., to components that mainly contribute to the flavor of food (Grosch, 2001; Bult et al., 2002; Reinhard et al., 2010); the odor of familiar/unfamiliar conspecifics (Breed and Julian, 1992); or more generally to pheromones (single odorants or associations of key odorants), which are sometimes carried in complex biological fluids or secretions (Schaal, 2010; Martin et al., 2013). A second strategy consists of attributing additional or unique information to the odorants forming a mixture as a whole, which carries a behavioral value that is distinct from the individual value of each component, i.e., to perceive the mixture as a single meaningful object (see previous section on odor objects and configural processing of odorants in mixture). This configural strategy is functional both in aquatic and terrestrial organisms. For instance, after food-rewarded exposures, catfish differentially modify their swimming activity in response to mixtures of amino-acids and to their elements (Valentincic et al., 2000, 2011). Spiny lobsters display food searching and exploration/avoidance responses that illustrate their ability to differentially process and perceive mixtures of odorants and odorants themselves (Fine-Levy et al., 1989; Lynn et al., 1994; Livermore et al., 1997). In a double-choice test, a mollusk, the terrestrial slug, displays a strong aversion to a binary mixture while the odor of each component remains strongly attractive (Hopfield and Gelperin, 1989). In insects, the configural perception of odor mixtures is involved in flower-foraging behaviors. For example, when exposed to flower-scents containing dozens of components, bees perceive certain mixtures of volatile molecular constituents as configurations, an ability that certainly contributes to the discrimination of flowers and expression of preferences for those offering higher quality or quantity of nectar (Deisig et al., 2001; Wright et al., 2009). In rats, the configural perception of odor mixtures influences their spatial performance, localization of reward, and digging activity related to foraging (Staubli et al., 1987; Linster and Smith, 1999). In dogs, and especially military dogs, the discrimination between complex mixtures of volatiles and their elements may be critical in the detection of explosives (Lazarowski and Dorman, 2014). In humans, odor mixture processing may support the categorization of food while simultaneously keeping the ability to differentiate between different products that belong to the same category due to the perception of inconstant elements in addition to invariant configurations (Gottfried, 2009).

The chemical environment is complex not only for adult organisms but also for young, neonates, fetuses, and embryos, even if it is more limited during earlier periods of development (e.g., when the organism is developing in the maternal body, nests, or eggs). Indeed, maternal fluids such as amniotic fluid, colostrum, or milk in mammals, and more generally the maternal body itself, generate or carry a large number of odorants (Antoshechkin et al., 1989; Schaal, 2010). Very young organisms have an urgent need to respond to some of these odors to rapidly interact with the mother; to localize the nipples and suck; and to expand their knowledge about the surroundings. Interestingly, although this remains to be more generally investigated, both elemental and configural processing appear functional early in life. Thus, newborn rabbits respond to the monomolecular mammary pheromone (2-methylbut-2-enal) carried in milk among 150 other odorants (Coureaud, 2001; Schaal et al., 2003; Coureaud et al., 2010), and they elementally process “artificial” mixtures containing up to six components. They are also able to perceive configurations in some binary and senary mixtures (Coureaud et al., 2008, 2010, 2011; Sinding et al., 2011, 2013). As in adults, the ability of very young organisms to process odor mixtures both configurally or elementally may contribute to decision making and to the discrimination between a peculiar conspecific, the mother, which carries peculiar odor elements or definite configurations, and another category of conspecifics, the lactating females, which emit the same or at least overlapping elements and configurations (Coureaud et al., 2006, 2011; Logan et al., 2012).

## IMPLICATIONS OF ODOR MIXTURES PROCESSING IN INDIVIDUALS WITH MOOD DISORDERS

Olfactory dysfunction may be a prodrome of neurodegenerative diseases such as Alzheimer’s and Parkinson’s disease (Albers et al., 2006; Djordjevic et al., 2008). Because of the partial overlap between the brain structures involved in affective disorders, olfaction and emotion, olfactory impairments can be observed in several psychiatric diseases: major depression (Pause et al., 2001; Atanasova et al., 2010), seasonal affective disorder (Postolache et al., 1999), anorexia nervosa (Kopala et al., 1995), psychoses (Moberg and Turetsky, 2003), and obsessive compulsive disorder (Hermesh et al., 1999). These impairments affect different aspects of olfactory function (i.e., detection threshold, odor identification, discrimination, memory, intensity, familiarity, and pleasantness) and depend on the nature and extent of psychiatric and neurological involvement.

The majority of olfactory studies and mood disorders have focused on the perception of single odorants. To date, only a few studies have investigated olfactory perception in major depression using odor mixtures (Atanasova et al., 2010; Atanasova, 2012; Naudin et al., 2012). However, studies using odor mixtures are of specific interest because complex olfactory stimuli reflect daily life situations, which is important in the study of anhedonia (failure to gain pleasure from normal pleasant experiences). Anhedonia is considered as a core symptom of major depression in an objective way. Using binary mixtures of both pleasant (vanillin) and unpleasant (butyric acid) odorants at three different iso-intense concentrations, it has been shown that depressed patients perceived the majority of odor mixtures (67%) as significantly less pleasant compared to healthy subjects (Atanasova et al., 2010; Atanasova, 2012). Depressed subjects also had low performance in correctly identifying the odor of the odorants within the binary iso-intense mixture, and they more readily perceived the unpleasant compound compared to control subjects. The perception of a binary odor mixture depends on the subjects’ psychological state and depressed level; a higher depression score is associated to a better perception of the unpleasant stimulus and to a lesser perception of the pleasant stimulus within a binary iso-intense mixture (Atanasova et al., 2010). These observations were confirmed and generalized in a study using an iso-intense mixture of another pleasant (2-phenylethanol) and unpleasant (isovaleric acid) odorant (Naudin et al., 2012). Since the same results were obtained in patients during a depressive episode and in remission, the authors suggested that these olfactory impairments may constitute potential trait markers of depression. These results could be explained by the cognitive bias for emotionally negative stimuli observed in depression that could persist in the remitted state (Bhalla et al., 2006).

All of the observations revealed that anhedonia can be advantageously observed in depressed patients at the olfactory level with complex olfactory stimuli. They also suggest that the loss of food cravings often described in depression could be partly explained by a modification in olfactory perception, ending in a better perception of unpleasant sensory components in food. This finding emphasizes the importance of using complex mixtures of odorants, which are more ecologically relevant stimuli, to better understand the modulation of olfactory perception in mood disorders. Future psychophysical, neurophysiological, and neuroimaging investigations are needed in this field to increase our knowledge of the etiology of the diseases and to develop the appropriate tools to better care for patients with affective disorders.

## IMPLICATIONS OF ODOR MIXTURES PROCESSING IN ODOR STIMULI FORMULATION

Odors (orthonasal smell and retronasal aroma) are key perceptual characteristics to formulate in foods and in home and personal care products. It is the first chemical sense involved when a consumer is using such a product. Consumers base their opinion on the quality of a product, i.e., whether they like it and whether it is fulfilling its intended function, based partly (for food products) or completely (for perfumes) on the olfactory experience. Therefore, formulating the right olfactory experience cannot be taken lightly. Most food and beverage companies employ the services of flavor companies to create the flavors or aromas that will enter the formulation of the end product. Indeed, food and beverage companies may require flavors for their new products or for compensating changes in the formulation of their existing products.

Focusing on olfactory perception, which is largely involved in flavor (Hornung and Enns, 1989; Thomas-Danguin, 2009), we explained in the previous sections of this review that odors arise from perceptual representations of mixtures of odorants, whose construction is far from being fully understood and remains mostly impossible to predict on the basis of chemical composition. Within flavor houses, flavor formulation is thus performed by specially trained scientists called flavorists, who have empirical knowledge about the perception of chemicals in mixtures. They know a large variety of odorous raw materials but also specific mixtures’ recipes to produce specific flavors and continuously create new ones. Usually, they follow a brief delivered by the client. This brief must specify the direction of flavor to be formulated (e.g., strawberry), the type of product into which the new flavor will be incorporated in (e.g., dairy product), and other requirements (e.g., all natural). It is then the role of the flavorist to use his/her expertise with the chemical ingredients at his/her disposal and his/her experience to formulate a flavor mixture that match the client’s requirements. The flavor house may also seek the assistance of an application specialist to ensure that the newly formulated flavor will deliver its expected quality in the application for which it is intended. Indeed, when formulated in a complex matrix, such as a food matrix (e.g., a chocolate bar), interactions with the different components of the matrix can influence the volatility of the odorants within the mixture and, consequently, the whole headspace mixture composition (Guichard, 2002).

In perfume composition, creation also relies on empirical knowledge. For instance, it is known that adding sulfur components, which are often unpleasant (e.g., cat urine odor), could give a lift to a fruity component in a complex mixture of odorants evoking a tropical fruit odor. Indeed, we have presented several examples of the impact of an unpleasant odor mixed with a pleasant one. Synergistic effects are also extensively used in perfume design. For instance, fatty aldehydes are known to enhance many floral odors at low concentrations, even if their own odor is very different from the target one. These synthetic odorants have been used in floral-aldehydic perfumes such as the famous Chanel no.5 created by Ernest Beaux for the house of Chanel in 1921 (Chastrette, 1995). Perfume chords are also very well empirically used in this industry. The concept of perfume chords is reconcilable with configural processing of odor mixtures. Indeed, chords usually rely on mixtures of three or four odors (which are sometimes linked to pure chemicals) that are included in larger formulae. This is made possible by perfumers after a huge amount of trials following the artist’s intuition (Chastrette, 1995). Moreover, as explained by the famous perfumer Edmond Roudnitska (quoted by Chastrette, 1995), a perfume composition includes not only one chord but an unknown number that are not smelled one after the other but can overlap, be enhanced, or be canceled. Therefore, the perceptual interactions that result from smelling a perfume are likely the playground of the artist and allowed him to create esthetic odor objects.

Besides the complexity of formulating a flavor or a perfume based on product properties, top-down influences also play a role in the way consumers perceive a product. Indeed, packaging (color, shape) and the type of claim made on the product can influence the consumer’s perception of the product (e.g., Gatti et al., 2014). Finally, the above examples demonstrate the empirical knowledge and methods used in the formulation of aromas and fragrances but also describe how recent insights into odor processing and perception impact the development of new products.

## CONCLUSION

The study of odor mixtures is an original window to investigate olfactory processes in a manner that may be more relevant to ecological perceptual contexts, which is crucial to understanding how organisms, including humans, represent and adapt to their chemical complex environment. It is also an original path to identify, characterize and further treat adaptation disorders in humans.

However, it is obvious that the scientific knowledge available on odor mixtures’ perception, even the simplest ones with only two odorants, is far from being up to empirical knowledge. Yet, a better understanding of the underlying biological processes involved when organisms manage to identify an odor object based on hundreds of chemicals in a few milliseconds would likely impact many scientific fields. Indeed, deciphering what odors (elements and/or configurations) are perceived in a mixture may contribute to the efficiency of flavor analysis, the identification of key components of food acceptance or disliking, and the elaboration of food flavors and perfumes. Moreover, extending our investigations on the odor processing of natural mixtures would shed light on the ability of organisms, including humans, to code complex information in the olfactory brain and how, through development, learning, or evolution, the resulting odors are stored as perceptual objects and reused by individuals.

It appears from this review that the appropriate description of the stimulus representations is likely the most critical factor in odor mixture perception. This is fundamental and should not be overlooked since a mixture is not a simple addition of each of its component and because it is the starting point of every following process. This requires for a large part to clearly pinpoint the peripheral spatiotemporal coding processes of odorants in mixtures, which is the only way to decipher the role of mixture composition and to predict accurately odor perception on the basis of chemical composition. Nevertheless, the incoming information is highly subjected to modulations at all stages of integration. If we highlighted in this review that the processing is contrasted at each stage, the specific role of these distinct stages remains largely to be discovered. To take up these research challenges, one should favor a systemic approach that would combine several investigation levels thus gathering cellular, neurobiological and psychological aspects both in human and other animal species. That was the guideline of this review to put together the results obtained in various models in order to underline similitude and differences in perception mechanisms. Indeed multidisciplinary studies may help to tackle specific questions regarding both odor mixture coding and perception, plasticity of perception and behavioral consequences, and thus would likely bring the field forward.

## Conflict of Interest Statement

The authors declare that the research was conducted in the absence of any commercial or financial relationships that could be construed as a potential conflict of interest.
